# P-1224. Individual Target Pharmacokinetic/Pharmacodynamic Attainment Rates among Cefepime-treated Patients Admitted to the ICU with Hospital-Acquired Pneumonia with and without ECMO

**DOI:** 10.1093/ofid/ofae631.1406

**Published:** 2025-01-29

**Authors:** Adrian Valadez, Marc H Scheetz, Michael N Neely, Paul R Yarnold, Mengjia Kang, Helen K Donnelly, Kay Dedicatoria, Rachel Medernach, Sophia Nozick, Alan R Hauser, Egon A Ozer, Estefani Diaz, Alexander V Misharin, Richard G Wunderink, Nathaniel J Rhodes

**Affiliations:** Midwestern University, Downers Grove, Illinois; Midwestern University, Downers Grove, Illinois; The Saban Research Institute, Children’s Hospital Los Angeles, University of Southern California, Los Angeles, CA, USA, Los Angeles, California; Optimal Data Analysis LLC, Chicago, Illinois; Northwestern University, Chicago, Illinois; Northwestern University, Chicago, Illinois; Midwestern University, Downers Grove, Illinois; Northwestern University Feinberg School of Medicine, Chicago, Illinois; Northwestern University, Chicago, Illinois; Northwestern University, Chicago, Illinois; Northwestern University Feinberg School of Medicine, Chicago, Illinois; Northwestern University, Chicago, Illinois; Northwestern University, Chicago, Illinois; Northwestern University Feinberg School of Medicine, Chicago, Illinois; Midwestern University, Downers Grove, Illinois

## Abstract

**Background:**

The impact of extracorporeal membrane oxygenation (EMCO) on cefepime (FEP) pharmacokinetics (PK) in the absence of renal replacement therapy (RRT) is unclear.

Figure 1.

**Figure:**
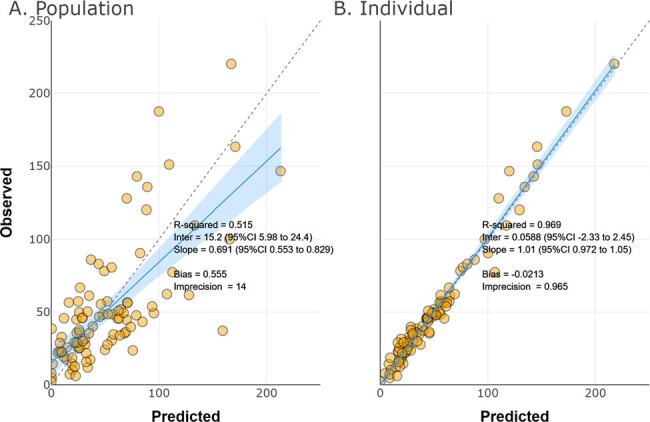

Observed versus predicted cefepime plasma concentrations in the population (A) and for each individual (B)

**Methods:**

We evaluated FEP PK in patients with hospital-acquired pneumonia from June 2018 to May 2023, with/without ECMO in the absence of RRT, in a single-center study nested within the Successful Clinical Response In Pneumonia Treatment (SCRIPT) study. Patient data were extracted from electronic health records. FEP dosing was according to institutional protocols based on renal function and indication. Plasma concentrations were quantified by validated LC-MS/MS assay. We used Pmetrics 2.1.1 for R to estimate population model parameter values [e.g., volume (V) and clearance (CL)] and individual target attainment rates, assuming a free (*f*) fraction of 80%. Targets of 100% *f*T_>1xMIC_ and 100% *f*T_>4xMIC_ were evaluated *vs*. the susceptible breakpoint MICs of Enterobacterales (2 mcg/mL) and *P. aeruginosa* (8 mcg/mL) and stratified by concurrent ECMO.

Figure 2.

**Figure:**
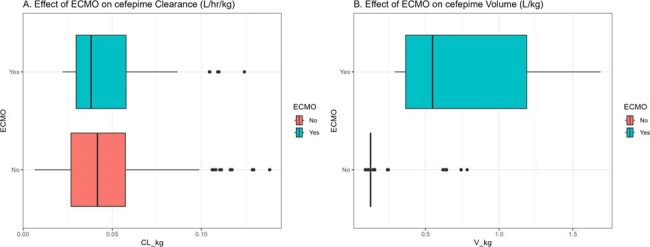

Pharmacokinetic parameters (CL and V) standardized to body weight (kg) stratified by ECMO status.

**Results:**

Sixty-two SCRIPT patients (63% male) contributed 95 plasma FEP samples (1-13 per patient). A total of 9 patients (one female) required ECMO. Patients had a mean±SD CRCL of 123±84 mL/min and a mean±SD body weight of 86±24 kg. The median/max first 24-hr FEP dose was 4/8 grams. A two-compartment PK model fitted best (Fig 1). Body weight and creatinine clearance were related to V and CL, respectively (Table 1). ECMO was not significantly associated with FEP CL (P=0.2) but was associated with V (P< 0.005) (Fig 2). ECMO patients had a median 3.5-fold greater Vd vs. non-ECMO patients. For targets of *f*T _>1xMIC_ and *f*T _>4xMIC_, median individual plasma attainment rates were 100% and 100% against Enterobacterales and 100% and 49% against P. aeruginosa, respectively. Attainment rates were lowest in patients requiring ECMO vs. P. aeruginosa at a target of 100% *f*T _>4xMIC_, (Fig 3).

Figure 3.

**Figure:**
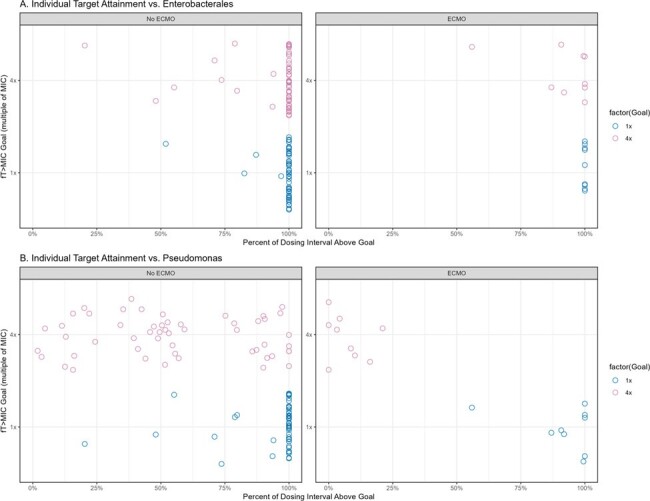

Individual predicted cefepime PK/PD target attainment stratified by organism breakpoint and concurrent ECMO status for fT>1xMIC and fT>4xMIC.

**Conclusion:**

We found that ECMO increased FEP V but not CL suggesting that ECMO patients may require loading doses and prolonged infusions to improve target attainment. Protocolized short infusions of FEP in ECMO patients resulted in suboptimal target attainment for a PK/PD goal of 100% *f*T_>4xMIC_ against *P. aeruginosa*. More work is needed to identify the impact of FEP target attainment on clinical outcomes in populations such as these.

Table 1.

**Figure:**
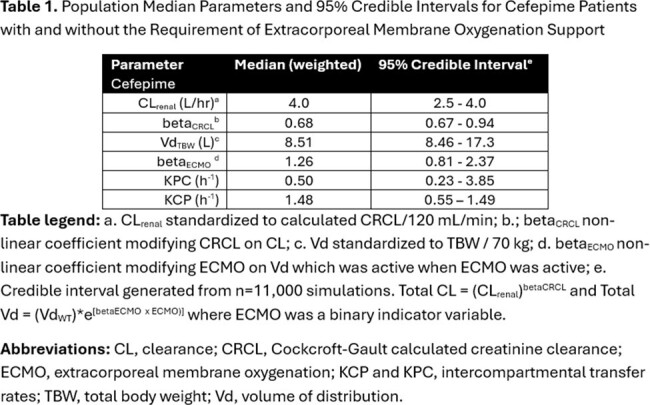

Population Median Parameters and 95% Credible Intervals for Cefepime Patients with and without the Requirement of Extracorporeal Membrane Oxygenation Support

**Disclosures:**

**Marc H. Scheetz, PharmD, MSc**, Abbvie: Advisor/Consultant|Basilea: Advisor/Consultant|Cidara: Advisor/Consultant|DoseMe: Advisor/Consultant|Entasis: Advisor/Consultant|F2G: Advisor/Consultant|GSK: Advisor/Consultant|Lykos: Advisor/Consultant|Roche: Advisor/Consultant|Third Pole Therapeutics: Advisor/Consultant|Xelia: Advisor/Consultant **Nathaniel J. Rhodes, PharmD MS**, Apothecademy, LLC: Advisor/Consultant

